# Utilization of health insurance by patients admitted for dental surgical procedures at a tertiary care hospital in Coastal Karnataka: a retrospective study

**DOI:** 10.12688/f1000research.139841.2

**Published:** 2023-11-30

**Authors:** Bhargav Bhat, Ramprasad Vasthare, Nishu Singla, Prajna P Nayak, Ashwini Kumar, Ritesh Singla

**Affiliations:** 1 Department of Public Health Dentistry, Manipal College of Dental Sciences, Manipal, Manipal Academy of Higher Education, Manipal, Karnataka, 576104, India; 2 Department of Community Medicine, Kasturba Medical College, Manipal, Manipal Academy of Higher Education, Manipal, Karnataka, 576104, India; 3 Department of Orthodontics and Dentofacial Orthopaedics, Manipal College of Dental Sciences, Manipal, Manipal Academy of Higher Education, Manipal, Karnataka, 576104, India

**Keywords:** Health Insurance, Dental insurance, Oral surgical procedures, Insurance schemes

## Abstract

**Background:** There are various medical insurance options available in India. However, unlike many other countries, dental insurance plans are rare. The aim of this study was to assess the utilization of various government and private health insurance schemes by patients admitted for dental surgical procedures at a tertiary care hospital in coastal Karnataka, India.

**Methods:** A study was conducted retrospectively to gather data on the socio-demographics, bill details, insurance, and benefits claimed by patients admitted to the Department of Oral and Maxillofacial Surgery at a tertiary care hospital from May 2016 to September 2022.

**Results:** Out of 1750 patients, only 856 (48.9%) patients had availed of insurance, 395 patients (22.6%) utilized government health insurance policies, and 461 patients (26.3%) availed of private health insurance plans. Among Government schemes, primarily Ayushman Bharat-Arogya Karnataka was used by 262 (30.6%) patients, followed by Employees’ State Insurance Scheme by 110 (12.9%) patients. Among private schemes, 212 (24.8%) patients used the policies purchased by them, 19 (2.2%) patients’ medical expenses were paid by their employers, 105 (12.3%) patients utilized Manipal Arogya Suraksha and 124 (14.5%) patients used Medicare provided by the hospital. Bivariate linear regression confirmed that the total bill amount, out-of-pocket expenditure by the patient, and insurance amount reimbursed to the hospital were significantly associated with the type of insurance (government vs. private). The study noticed a gradual rise in insured patients every year.

**Conclusion:** Greater utilization of health insurance should be encouraged because the cost of dental treatment has always hindered the use of oral health services worldwide. This study highlights that the benefit available to the patients were mainly through general health insurance schemes, not specifically dental health insurance. Insurance schemes covering dental must be promoted more aggressively in the media, highlighting their available benefits, merits, and demerits.

## Introduction

One of the basic rights of every individual is to be healthy. However, there is a constant rise in the cost of health care. The ultimate goal of healthcare systems worldwide is to ensure everyone has equal access to healthcare, irrespective of financial capacity. Health insurance is a feasible option for dealing with rising healthcare prices, increased demand for healthcare services, and availing good quality care by low-income groups. Today, there is plenty of general health insurance policies in India. The Insurance Regulatory and Development Authority (IRDA) bill implemented by the Indian government paved the way for the country’s development and overall supervision of the insurance sector.

The first significant social security legislation implemented by the Indian government was the Employee’s State Insurance Act (ESI Act) in 1948. It offers benefits for illness, maternity, and fatal workplace accidents. The Central Government Health Scheme (CGHS) for central government personnel, lawmakers, judges, liberation warriors, and their families was the second initiative introduced in India in 1954. To accomplish Universal Health Coverage (UHC), the Indian government has introduced the Ayushman Bharat program in 2018. The Pradhan Mantri Jan Arogya Yojana (PMJAY) and Health and Wellness Centers are the two essential parts of the Ayushman Bharat scheme (HWCs).

The State Government of Karnataka in South India introduced the Suvarna Arogya Suraksha Scheme in 2009. To oversee the program, the Suvarna Arogya Suraksha Trust was established and registered as an independent entity under the Department of Health and Family Welfare, adhering to the Indian Trust Act of 1882. Suvarna Arogya Suraksha Trust has been at the forefront of successfully implementing this healthcare program to serve a significant portion of BPL (Below Poverty Line) and APL (Above Poverty Line) families. The government defines BPL families as those with annual household income less than Rs. 15,000, while APL families have an annual household income between Rs. 15,000 and Rs. 1 Lakh. In Karnataka, this program is known as Ayushman Bharat Arogya Karnataka (AB-ArK). ^1^

Community-Based Health Insurance (CBHI) programs cover benefits based on a group premium for community groups generally run by charitable trusts or non-governmental organizations (NGOs). These can be a valuable alternative to universal health care in a developing nation like India, where considerable income and wealth discrepancies exist. ^2^ CBHI programs in Karnataka include Yashasvini, Karuna Trust, Manipal Arogya Suraksha scheme, etc. Moreover, there are some public or private sector employers, namely, the Indian Army and Public Sector Undertakings banks, which give reimbursement for treatments and employer-based insurance programs to their employees, such as the Medicare program in this hospital. In the Indian context, private health insurance mainly contracts with urban-based corporate hospitals. ^3^ However, a significant fraction of India’s population, 85.9% rural and 80.9% Urban, still cannot afford the private health insurance plans. ^2^^,^^4^^,^^5^

There are numerous medical insurance options in India nowadays. However, few insurance companies provide exclusive dental coverage under various general, health, and life insurance policies. Unlike most other countries, specific dental insurance plans are not standard in India. Even with the available options, routine dental ailments are usually left out, and only dental emergencies/accidents are usually covered. This may be because, apart from oral cancer, dental problems are usually considered non-life-threatening. ‘Pepsodent Dental Insurance’ discontinued today, launched by Hindustan Lever in partnership with ‘New India Assurance,’ the first dental insurance scheme in India. ^6^ ‘ICICI Lombard Dental Insurance Cover’ was one of the first plans under the general health insurance plan ‘Health advantage plus policy’ that reimbursed for dental consultation and some charges for out-patient treatments. ^6^^,^^7^ ‘Apollo DKV Health Insurance’ - ‘Easy Health Premium’ plan, also covers some dental treatment charges on an outpatient basis.

The Indian Dental Association has only partially successfully brought out a comprehensive Indian dental insurance scheme. Indian dental insurance plans are of two main types: - (1) Stand-alone dental insurance plan that covers the set amount of costs associated with general dental issues, including periodontitis and the removal of permanent teeth. Popular manufacturers of dental care products typically provide this package in collaboration with an insurance provider. (2) Dental insurance coverage as part of a general health insurance plan like the Health Advantage or Student Medical Policy. Dental costs might be claimed with other types of compensation, such as the expense of medication or hospitalization. ^8^ However, people can get prophylactic and preventative care if dental insurance is available. It can assist in easing the burden of oral disease and lowering the cost of dental care. ^9^

However, ‘fee for service’ is still the primary payment system for dentists in India. Here people primarily seek curative dental care services, and preventative measures are not given much importance. Also, the utilization of dental services by people has remained low due to the high cost of dental treatment. ^10^ Moreover, oral health is usually ignored as general health is prioritized while purchasing insurance-covering medical expenses. Since there is a scarcity of studies on dental health insurance in the Indian scenario, an attempt is made here to assess the utilization of various government and private health insurance schemes and trends in the sociodemographic profiles among insured and uninsured patients admitted for oral surgical procedures under the Department of Oral and Maxillofacial Surgery at a tertiary care hospital in coastal Karnataka.

## Methods

A retrospective study was conducted at Kasturba Hospital, Manipal, in September 2022. The study methodology was thoroughly reviewed and approved by the Institutional Ethics Committee (IEC 748-2020). Due to the retrospective nature of the study involving data collection on anonymized medical records, patient consent was waived. Inclusion criteria of the study was to include the data of only those patients who were admitted for various oral surgical procedures at Kasturba Hospital.

The conditions such as cysts, potentially malignant disorders, benign and malignant tumors, and salivary gland disorders were categorized as cysts and tumors. Road traffic accidents and other trauma cases were classified as facial fractures. Osteomyelitis, clefts, orthognathic surgeries, and space infections were categorized under major surgeries performed under general anesthesia. Extraction, impaction, and post-extraction complications, such as oronasal fistula, were categorized under minor surgical procedures.

This study collected data of all patients admitted from May 2016 to September 2022 under the Department of Oral and Maxillofacial Surgery at Kasturba Hospital, Manipal. Incrementally and periodically, 20-30 medical records per day were accessed at the Medical Records Department of the hospital with due efforts to maintain the patient’s anonymity following proper infection control, sanitization, and social distancing as per the rules and regulations of the hospital.

The following sociodemographic data were collected, i.e., age, gender, admission and discharge date, religion, district, and state of residence. The patients’ ages were categorized into groups 0-16, 17-30, 31-45, and 46 and above. The following financial data were obtained from the Department of Finance of the hospital: the details of the bill produced by the hospital, the type of insurance claimed by the patient, the amount of monetary benefit claimed by the patient, and out-of-pocket payment made at the time of discharge.

The present study categorized the insurance claims under uninsured, government, and private health insurance plans. Employee’s State Insurance Act (ESI), Central Government Health Scheme (CGHS), universal coverage (Ayushman Bharat- Arogya Karnataka), and other state government insurance schemes were grouped under government insurance. Third-Party Administrator (TPA), Voluntary Health Insurance, community health insurance, micro health insurance, employer-based health insurance, and Medicare were grouped under private health insurance schemes. Out-of-pocket expenditure is the amount of cash paid by the patient at discharge, apart from the reimbursed amount by health insurance and/or the entire amount paid by the patient without insurance coverage.

### Statistical analysis

The data obtained was first entered into a Microsoft Excel sheet. The statistical analysis was performed using SPSS version 24.0 (IBM Corp). Sociodemographic of the study population were compared between insured and uninsured patients using the chi-square test. The trends in the utilization of Health insurance from 2016 to 2022 were evaluated with descriptive statistics. The results were presented in frequency tables and graphs.

One-way ANOVA followed by Tukey’s Post hoc analysis was run to compare the mean bill amount, out-of-pocket payment by the patient, and reimbursed insurance amount towards the hospital among uninsured, government-insured, and privately-insured patients. These results were further confirmed using bivariate linear regression with the mean bill amount, the out-of-pocket payment made by the patient, and insurance reimbursed towards the hospital as the dependent variables and type of Insurance (Government vs. private) as the independent variable.

## Results

**Figure 1.**
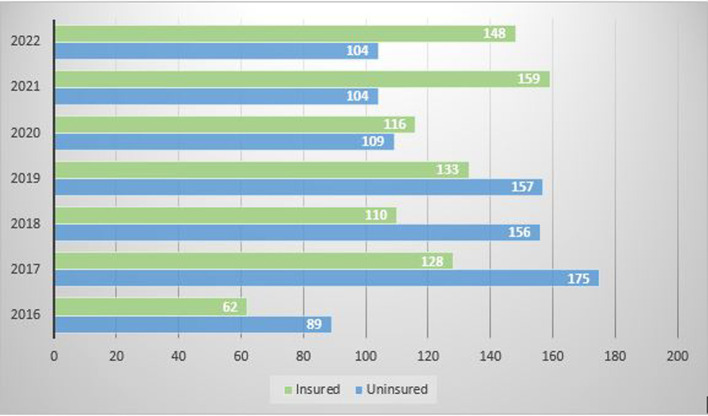
Admissions per year under oral and maxillofacial surgery showing the number of insured and uninsured patients.

The total medical records of 1750 patients admitted to the hospital under the Department of Oral and Maxillofacial Surgery from May 2016 to September 2022 were retrieved in the present study. It was found that only 856 (48.9%) patients, out of 1750 patients, had availed of insurance at the time of discharge, and 894 (51.1%) patients were uninsured. It was observed that out of the total number of admissions in 2016, 41% were insured; in 2017, 42.2%; in 2018, 41.4%; in 2019, 45.9; in 2020, 51.5%; in 2021, 60.5%; whereas in the year 2022 till September, there were 58.73% of already insured patients ( Figure 1). Thus, suggesting a gradual rise in the percentage of insured patients.

The male patients admitted were significantly higher than the female patients in the present study (p<0.001). Among males, 656 (49.3%) of 1331 patients were insured, and among females, 200 (47.7%) out of 419 patients were insured. It was noticed that the admissions for the pediatric age group of 0-16 were only 4.4%, whereas it was highest among the 17-30 years age group, followed by 29.2% among 46 and above and 26.2% among the 31-45 years group. Among the Christians, 59.4%; Hindus, 49.3%; and Muslims, 38.7% were insured. Based on the area of residence, our study showed that 531(57.7%) out of 921 patients from the Udupi district were insured, and 325 (39.2%) out of 829 patients from other districts across the state and country were insured. Also, it was seen that 796 (48.2%) of 1651 from Karnataka were insured, and 60 (60.6 %) of 99 patients from other states were insured ( Table 1).

**Table 1.** The sociodemographic distribution between the uninsured and insured study groups.

Variables		Number of patients	Uninsured	Insured	P value
Gender	Male	1331	675 (50.71%)	656 (49.28%)	**<0.001** *
	Female	419	219 (52.26 %)	200 (47.73%)	
Age	0-16	77	46 (59.74%)	31 (40.26%)	0.151
	17-30	702	341 (48.58%)	361 (51.42%)	
	31-45	459	233 (50.76%)	226 (49.24%)	
	46 and above	512	274 (53.52%)	238 (46.48%)	
Religion	Christian	64	26 (40.62%)	38 (59.37%)	**<0.001** *
	Hindu	1562	792 (50.7%)	770 (49.29%)	
	Islamic	124	76 (61.29%)	48 (38.7%)	
District	Udupi	921	390 (42.3%)	531 (57.7%)	**<0.001** *
	Other	829	504 (60.8%)	325 (39.2%)	
State	Karnataka	1651	855 (51.78%)	796 (48.21%)	**<0.001** *
	Other states	99	39 (39.39%)	60 (60.6%)	
	Total	1750	894 (51.1%)	856 (48.9%)	

* Statistical significance set at p ≤ 0.05.

**Figure 2.**
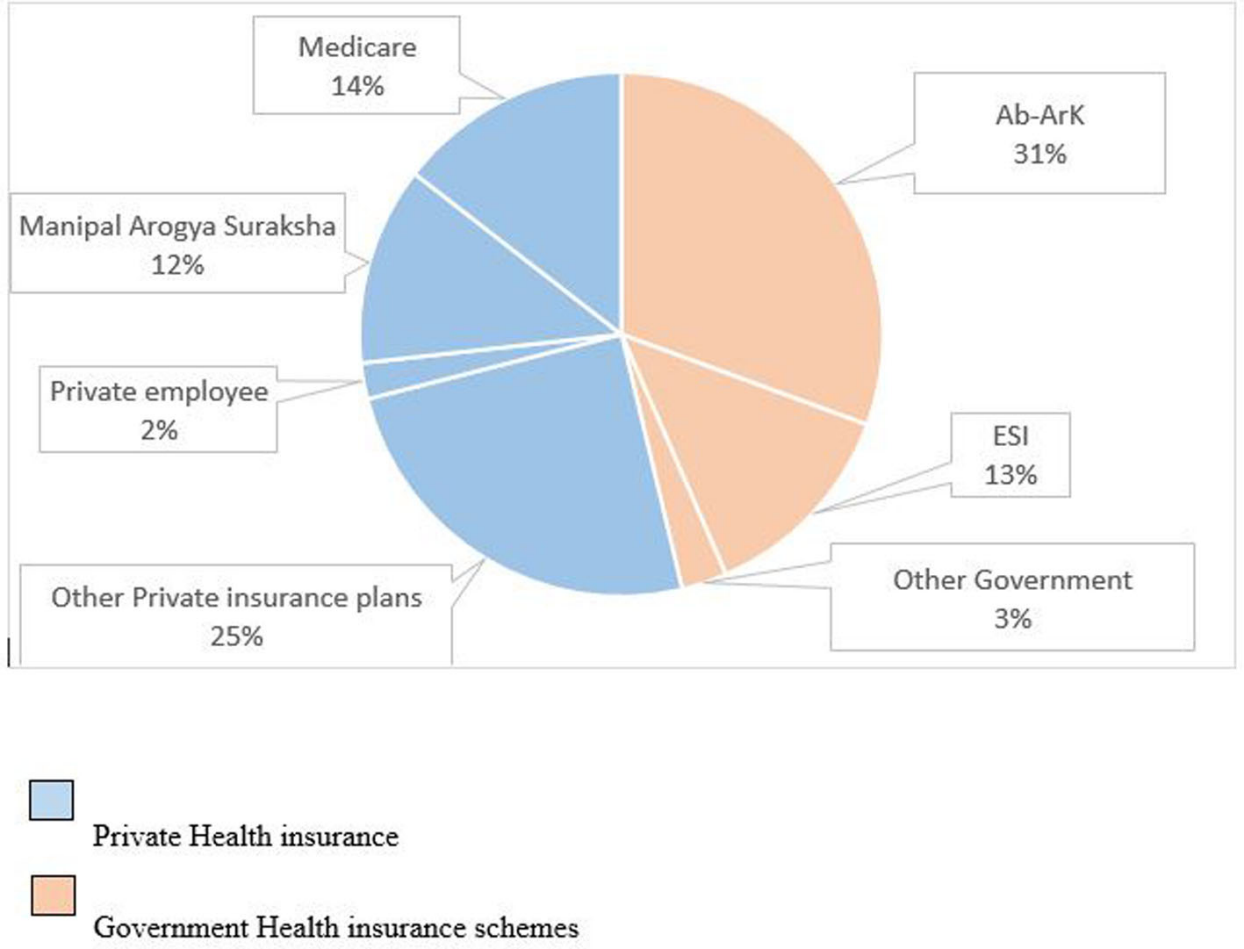
Distribution of type of insurance schemes claimed by the insured patients.

According to study results, 395 patients (22.6%) have utilized Government Health cover schemes, and 461 patients (26.3%) have availed of private Health insurance plans. Among the Government Insurance schemes, primarily the Universal Health Insurance of India named Ayushman Bharat- Arogya Karnataka was utilized by 262 (30.6%) patients, followed by the Employees’ State Insurance Scheme utilized by 110 (12.9%). The other Government insurance schemes used by patients were the Mukhyamantrigala Santwana Harish plan (1.6%), Arogya Bhagya Yojana by the National Accreditation Board for Hospitals & Healthcare Providers – NABH (1%), and Snehasantwanam – rehabilitation of endosulfan (0.1%). Among the Private health insurance schemes, it was observed that 212 (24.8%) had used the policies purchased by them for the coverage of their medical expenses. While 19 (2.2%) patients’ medical expenses were paid by their employers, 105 (12.3%) patients were covered under Manipal Arogya Suraksha and 124 (14.5%) patients utilized Medicare provided to the students and employees of the hospital ( Figure 2, Table 2).

**Table 2.** Details of the insurance schemes availed by all the study patients.

Name of the insurance	Frequency	Percentage	Type of insurance
No insurance – Cash payment	894	51.1%	
**Government insurance**			
Arogya Karnataka APL	8	0.5%	Ayushman Bharat-Arogya Karnataka
Arogya Karnataka-BPL	254	14.5%	
Employee's State Insurance (ESI)	110	6.3%	Employee's State Insurance
Mukhyamantrigala santwana harish scheme	14	0.8%	Government (others)
Arogya Bhagya yojane - NABH	9	0.5%	
Snehasantwanam-rehabilitation of endosulfan	1	0.1%	
**Private insurance**			
Sampoorna Suraksha	92	5.3%	Private insurance companies
TTK healthcare services private	5	0.3%	
Star Health and allied insurance co ltd	19	1.1%	
Medi Assist India private ltd	28	1.6%	
HDFC	3	0.2%	
Vidal Health Pvt ltd	5	0.3%	
Dedicate health care service private ltd	1	0.1%	
Apollo Munich health insurance	2	0.1%	
Raksha health insurance Pvt ltd	1	0.1%	
Religare health insurance Pvt ltd	1	0.1%	
ICICI Lombard general insurance	3	0.2%	
Manipal Cigna – Suraksha scheme	34	1.9%	
Nuclear power corporation ltd	7	0.4%	
Iffco-tokio general insurance company ltd.	1	0.1%	
Bajaj allianz general insurance co. ltd	3	0.2%	
Care health insurance company ltd	1	0.1%	
Future General India Insurance co ltd	1	0.1%	
United Healthcare India	2	0.1%	
Universal Sompo General Insurance co ltd	3	0.2%	
Others (private insurance)	7	0.4%	
Konkan railway - Karwar	5	0.3%	Employer-based insurance schemes: offered by companies to their employees
Kudremukh iron ore company limited	1	0.1%	
Integrated tribal development program (ITDP)	3	0.2%	
Karnataka Fire & emergency service	1	0.1%	
Mangalore Refinery and petrochemicals ltd	1	0.1%	
ECHS polyclinic Mangalore	1	0.1%	
G Shankar blood donor card	7	0.4%	Community-based health insurance
Manipal Arogya Suraksha Nonkonkani	41	2.3%	
Manipal Arogya Suraksha RMCWH	7	0.4%	
Manipal Arogya Suraksha Konkani	50	2.9%	
Mahe Medicare	124	7.1%	MAHE Medicare
Total Insured patients	856	48.9%	

The results of One-way ANOVA show a statistically significant difference in total bill amounts (F=37.65; P=0.001), out-of-pocket payment by the patient (F=172.3; P=0.001), and insurance coverage amount (F=280.58; P=0.001) between government and privately insured patients. Tukey’s Post hoc analysis displays a significantly higher mean bill amount paid by government-insured patients (Rs. 64, 216) compared to privately insured type (Rs. 51924) and non-insured (Rs. 39807). The privately insured patients’ mean bill amount was significantly higher than the non-insured group (Rs. 39807). The out-of-pocket expenses paid by private insurance type Rs. 11938 (out of Rs. 51924) were considerably higher than the government-insured patients’ Rs. 4171 (out of Rs. 64, 216). However, the insurance coverage amount was significantly higher by the private insurance companies than the government insurance (P < 0.001) ( Table 3).

**Table 3.** Comparison of mean Bill amount (F=37.65; P=0.001), Out of pocket payment from the patient (F=172.3; P=0.001), and Insurance coverage amount (F=280.58; P=0.001) among Non-insured, Government insured and Private insured type.

		One way ANOVA					Tukey’s posthoc analysis		
		N	Mean	SD	F	P Value	Multiple Comparison	Mean Difference	P Value
Total Bill amount at the time of discharge	Non-insured **(A)**	894	39806.7	44185.5	37.657	<0.001 *	A vs. B	-24409.6	<0.001 *
	Government **(B)**	395	64216.3	47684.3			A vs. C	-10920.0	<0.001 *
	Private **(C)**	461	50726.7	51923.7			B vs. C	13489.6	<0.001 *
Out-of-pocket- expenditure	Non-insured **(A)**	894	39087.6	43656.5	172.306	<0.001 *	A vs. B	34916.8	<0.001 *
	Government **(B)**	395	4170.9	17108.3			A vs. C	27149.3	<0.001 *
	Private **(C)**	461	11938.3	27255.5			B vs. C	-7767.4	0.004 *
The insurance coverage amount	Non-insured **(A)**	894	0.0	0.0	280.583	<0.001 *	A vs B	-22958.5	<0.001 *
	Government **(B)**	395	22958.5	30077.4			A vs. C	-31782.0	<0.001 *
	Private **(C)**	461	31782.0	40121.3			B vs. C	-8823.5	<0.001 *

* Statistical significance set at 0.05; **N:** Number of samples; **SD:** Standard deviation.

These results were further confirmed using bivariate linear regression found that the total bill amount (β = 0.122; P = 0.001), out-of-pocket expenditure by the patient (β = -0.335; P = 0.001), and insurance coverage amount (β = 0.483; P = 0.001) were significantly associated with the government/private type of insurance. Moreover, the R ^2^ = 0.015 and 0.483 model explains that there was not much variance (1.5%) for the total bill amount but a significantly higher variance (48.3%) for the insurance coverage amount between government and private insurance. Also, the R ^2^ = 0.335 depicts 33.5% of the variance in out-of-pocket expenditure by the patients between government and private. It was noticed that the out-of-pocket expenditure of privately insured patients was 23.5% of the total bill compared to 6.5% of that of the government ( Table 4).

**Table 4.** Linear regression with Total Bill Amount, out-of-pocket expenditure by the Patient, and Insurance coverage amount as the dependent variables and Type of Insurance (government vs. private) as the independent variable.

Hypothesis	Regression weights	R Square	Std. Error	Beta Coefficient	P Value
H _1_1	Type of Insurance **➔** Bill Amount	0.015	0.028	0.122	**0.001** *
H _1_2	Type of Insurance **➔** Out-of-pocket expenses paid by the patient	0.335	0.022	-0.335	**0.001** *
H _1_3	Type of Insurance **➔** Insurance coverage amount by the Insurance scheme	0.483	0.02	0.483	**0.001** *

* Statistical significance set at 0.05; H _1_: Alternate Hypothesis.

H _1_ 1: There is a significant impact of the type of Insurance levels on the total Bill Amount. H _1_ 2: There is a significant impact of the type of Insurance levels on the amount paid by the patient (out-of-pocket expenditure). H _1_ 3: There is a significant impact of the type of Insurance on the Insurance amount reimbursed by the insurance company to the Hospital. Beta means standardized partial regression coefficient.

Type of Insurance: Government = 1, Private = 2.

## Discussion

This study assessed the utilization of various government and private health insurance schemes among insured and uninsured patients admitted for dental treatment at a tertiary care hospital in coastal Karnataka. Literature confirms the availability and utilization of dental insurance coverage in developed countries, like in Austria, compulsory health insurance schemes provide health coverage to around 99 percent of the population ^11^; in the U.K., dental care has been included under the National Health Service, which is a state-financed public oral healthcare system and is primarily funded through general taxation ^12^ and dental health insurance in the USA is regulated by the American Dental Association. ^13^ Still, such data reporting dental insurance coverage in India is scarce.

This may be because of the unpopularity of health insurance schemes covering dental treatments in India. Also, there is still a lack of awareness regarding the benefits of health insurance among the Indian population. Moreover, many people don’t generally tend to avail of health insurance, especially dental insurance, because dental health is neglected. ^14^ Hence, fee-for-service has remained a significant mode of payment for dental services in India. The present study revealed that only 48.9% of admitted dental patients were covered under insurance schemes, and around 51.1% had borne the expenses out of pocket. Only 22.6% of the study population availed dental benefits through government health insurance schemes and 26.3% through private health insurance schemes. This study supports that most Indian families usually don’t have any security or insurance to help them pay for their medical costs. Unfortunately, many families or individuals end up paying for health services out of their pockets. According to a study, most Indians spend their money on medical expenses and prescription purchases. ^9^

A cross-sectional study conducted in a private dental college and hospital among dental outpatients in Bengaluru reported that patients need better dental insurance awareness. In their research, among the insured patients, only 5% reportedly were aware of dental insurance integrated with general health insurance schemes. ^9^ Also, the utilization of dental services by people has remained low due to the high cost of dental treatment. ^15^ However, introducing dental insurance plans would reduce the financial burden and encourage individuals to take treatment early, making services available for all, thus reducing inequality. ^16^ The “National Health Interview Survey” in the USA has reported that dental coverage by insurance is the single most significant factor in determining whether a person sees a dentist.

The study participants were insured under five government and thirty private health insurance schemes. The Government insured participants were covered mainly under the universal health coverage of India, the Ayushman Bharat – Arogya Karnataka (Ab-ArK), followed by another essential social insurance scheme, i.e., the ESI Scheme. The privately insured participants were covered mainly by Manipal Arogya Suraksha, followed by private insurance schemes such as Sampoorna Suraksha, Manipal Cigna – Suraksha scheme, Medi Assist India Private Ltd, and Star Health and allied insurance Co. Ltd. In addition, Medicare was utilized by many students and employees of the hospital. This study highlights that most insurance schemes claimed by the study subjects admitted under the oral surgery department were general health insurance schemes, not specifically dental health insurance. This is because, unlike most Western countries, specific dental insurance plans are not standard in India. Here, oral health is usually integrated with general health insurance schemes. Additionally, it should be noted that some comprehensive dental insurance plans may not have been utilized by the individuals included in the study, as outpatients were not considered.

According to the present study, there was an almost similar distribution of insured and uninsured among the genders, ages, and religious statuses. However, it was observed that there were significantly higher males treated in the oral surgery department. This reflects that more male patients are being treated for oral cancer and trauma. A more significant percentage of people from the Udupi district claimed insurance than those from other districts. This is expected as most patients from the Udupi district and fewer from other districts visited this hospital because of its proximity. However, people from other districts who had availed of the treatment were either referred patients or those who didn’t have such hospitals near their residential areas. Noticeably, according to the state of residence, more people claimed insurance from other states. This may be mainly because of the utilization of Medicare facilities by the students or employees working for this hospital from various states.

In the present study, it was noticed that there is a rising trend in the utilization of health insurance schemes by patients, especially after the COVID pandemic (2020). This could be because of rising awareness of the benefits of the various insurance policies among people after the pandemic. This may also be because several foreign insurance firms have invested in India during the last few years. India has become a potential market due to its increasing purchasing power, growing demand for healthcare, expanding competitive private healthcare market, and rising rates of diseases. The trend of greater utilization of health insurance should be encouraged because the cost of dental treatment has always hindered the use of oral health services worldwide. ^17^ This will lead to greater use of dental services and, thereby, better oral health of the population.

In the present study, it was observed that there was a higher amount of bills generated through government-insured patients than private. Government insurance plans are low-cost policies backed by the government, ensuring the underprivileged can access health insurance coverage. The benefit of insurance obtained by dental patients was mainly from the utilization of the Ayushman Bharat scheme by the patients admitted under the Department of Oral Surgery. This scheme covers various surgical procedures done under general anesthesia for oral cancer, trauma cases, etc., and it also includes the coverage of the management of malocclusion through wiring and full-mouth pediatric caries in this hospital. The benefit of insurance obtained in this study was mainly by the patients admitted under the Department of Oral Surgery. The utilization of this scheme for outpatients, in general, was unexplored. Hence, the study participants primarily used this scheme for surgical procedures such as managing oral cancer and trauma. However, further studies are recommended to explore the usage of this scheme for other procedures on an outpatient basis.

It was noticed that the out-of-pocket expenditure of privately insured patients was 23.5% of the total bill compared to 6.5% of that of the government. This is clearly because of comparatively less coverage of medical expenses, high premiums, and the copayment methods of various private health insurance policies. Whereas government health insurance plans often offer highly discounted premiums, mainly following the deductible method of payment that covers a significant amount of medical expenses. Similarly, reimbursement to the hospital through private insurance schemes was significantly higher, i.e., 63% of the total bill compared to 36% through the government. Private insurance plans provide access to the higher sum insured and advanced medical care, usually at a higher premium. This indicates that the hospitals are mainly benefiting through private insurance schemes. In contrast, government insurance helps people, especially the vulnerable and needy population with low socioeconomic status.

### Limitations

This study was conducted at a single tertiary care hospital. Hence, the results may not be generalized to other places. Due to the COVID pandemic from December 2019 to June 2022, there was a significant fall in overall patients’ admissions for dental treatments. Further, it was noticed that during the COVID era, patients prioritized getting their inevitable therapy at the earliest instead of utilizing health insurance. This study mainly focused on dental in-patients (admitted to the hospital) through the oral and maxillofacial surgery department using dental insurance schemes. Patients who received dental outpatient services or were admitted by other departments, such as implant or pediatric, were excluded from the study due to technical issues.

## Conclusion

The present study reports that only half of the admitted dental patients availed the benefits of various government and private health insurance schemes. There are several schemes reported in the present study that offer dental benefits. This study highlights that the benefit available to the patients were mainly through general health insurance schemes, not specifically dental health insurance. Ayushman Bharat – Arogya Karnataka was utilized by a maximum number of patients, followed by third-party private insurance plans. Introducing dental insurance plans would reduce the financial burden and encourage individuals to take treatment early, making services available for all, thus reducing inequality. It is essential to raise awareness among the public and healthcare providers or referral doctors about the availability of different schemes.

### Recommendations

This study recommends prioritizing dental insurance schemes rather than merging them with general health insurance schemes. It is highly recommended to raise the general population’s awareness and health care providers or referral doctors to utilize various methods for dental procedures. Insurance schemes covering dental must be promoted more aggressively in the media, highlighting their available benefits, merits, and demerits. Implementation of a National Oral Health Policy with a clear directive for the role of dental insurance schemes must be implemented at the earliest.

## Data Availability

Mendeley Data: Utilization of health insurance by patients admitted for dental surgical procedures at a tertiary care hospital in coastal Karnataka: A retrospective study, https://doi.org/10.17632/3fvgjyxj8w.1. ^18^ This project contains the following underlying data:
- Raw Data – Insurance study.xlsx Data are available under the terms of the Creative Commons Attribution 4.0 International license (CC-BY 4.0).
