# Supplementary material for: Utilization of health insurance by patients admitted for dental surgical procedures at a tertiary care hospital in Coastal Karnataka: a retrospective study
Source: F1000Res. 2023 Nov 30;12:959. Originally published 2023 Aug 10. [Version 2] doi: 10.12688/f1000research.139841.2 (PMC10995532; doi:10.12688/f1000research.139841.2)
Supplement: Peer review history

# Reviewer report: Reviewer response for version 2

**DOI:** 10.5256/f1000research.155963.r247469

**Published:** 2024-04-04

**Authors:** Gajanan D Velhal

**Affiliations:** 1 Department of Community Medicine, B K L Walawalkar Rural Medical College, Ratnagiri, India

Present study undertaken by Dr Bhargav Bhat et al at Manipal Academy of Higher Education, India, mainly focuses on utilization pattern of various insurance schemes by the patients admitted for various dental surgeries.  Utilization of health insurance so far has been limited to major illnesses and surgical procedures demanding lot of expenses on the part of the patients. However use of insurance packages for dental surgical procedures is not documented much. Present study has attempted to find out utilization of insurance schemes for dental surgical procedures by the patients admitted at Kasturba Hospital, Manipal,  located in coastal Karnataka in India. Study findings are based on review of records of patients undergoing surgical processes during the period  from May 2016 to September 2022. Around 50% (48.9%, 856/1750) of the eligible patients had used insurance packages, however how many did not use it in spite of being eligible by virtue of their existing policies,  is not commented anywhere, being a retrospective record based study. Study findings reveal that Government health insurances policies are used by relatively less number of patients as compared to private health insurance plans, which is obvious taking into account the profile of patients availing private health services, level of awareness among those who avail private health services and the advocacy of insurance policy agencies for patients availing private sector health services.  Authors are not in a position to state, the level of awareness among patients regarding availability of insurance packages for dental surgical procedures.

 Attempt on the part of the authors to focus,  on coverage of dental surgical procedures in health insurance is highly appreciated. Utilization of health insurance packages by the people is increasing at present times, however there is hardly any data on its use for dental procedures. Dental surgical procedures are complex, costly and are usually resorted mainly by oral cancer victims. Even though the packages cover costly dental surgical procedures, there is no wide publicity given to this aspects and hence the use remains limited. Present study confirms the same. It would be of an immense help to the patients undergoing dental surgical procedures, if wide publicity is given to the clients of health insurance policies, as well as to the respective hospital authorities where complex and costly dental surgeries are undertaken. Periodic assessment of utilization by undertaking serial cross sectional studies by interviewing the patients, along different time lines in future, and at  different places would be very helpful to monitor the trend in this respect, supportive to improving the utilization by dental surgical patients in long run.

Is the work clearly and accurately presented and does it cite the current literature?

Yes

If applicable, is the statistical analysis and its interpretation appropriate?

Yes

Are all the source data underlying the results available to ensure full reproducibility?

Yes

Is the study design appropriate and is the work technically sound?

Yes

Are the conclusions drawn adequately supported by the results?

Yes

Are sufficient details of methods and analysis provided to allow replication by others?

Yes

Reviewer Expertise:

NA

I confirm that I have read this submission and believe that I have an appropriate level of expertise to confirm that it is of an acceptable scientific standard.

# Reviewer report: Reviewer response for version 2

**DOI:** 10.5256/f1000research.155963.r227207

**Published:** 2024-01-19

**Authors:** Bharathi M Purohit

**Affiliations:** 1 Division of Public Health Dentistry Centre for Dental Education and Research, CDER-AIIMS, New Delhi, New Delhi, India

Thank you for submitting the revised manuscript. I appreciate the changes made by authors which strengthen the manuscript further for publication.

 However, it is encouraged that the authors reply to the comments pointwise (with page number), for a successful review process.

 The manuscript can be accepted in the present form.

Is the work clearly and accurately presented and does it cite the current literature?

Partly

If applicable, is the statistical analysis and its interpretation appropriate?

Partly

Are all the source data underlying the results available to ensure full reproducibility?

Partly

Is the study design appropriate and is the work technically sound?

Yes

Are the conclusions drawn adequately supported by the results?

Yes

Are sufficient details of methods and analysis provided to allow replication by others?

Partly

Reviewer Expertise:

Epidemiology, oral health inequity

I confirm that I have read this submission and believe that I have an appropriate level of expertise to confirm that it is of an acceptable scientific standard.

# Reviewer report: Reviewer response for version 1

**DOI:** 10.5256/f1000research.153150.r195863

**Published:** 2023-09-11

**Authors:** Bharathi M Purohit

**Affiliations:** 1 Division of Public Health Dentistry Centre for Dental Education and Research, CDER-AIIMS, New Delhi, New Delhi, India

The study highlights the need for increasing dental insurance coverage in India. While, it is an important topic for research there are certain areas in which the manuscript needs to be strengthened to be appropriate for indexing. I have attached a marked up PDF  highlighting the areas for strengthening it further.

Is the work clearly and accurately presented and does it cite the current literature?

Partly

If applicable, is the statistical analysis and its interpretation appropriate?

Partly

Are all the source data underlying the results available to ensure full reproducibility?

Partly

Is the study design appropriate and is the work technically sound?

Yes

Are the conclusions drawn adequately supported by the results?

Yes

Are sufficient details of methods and analysis provided to allow replication by others?

Partly

Reviewer Expertise:

Epidemiology, oral health inequity

I confirm that I have read this submission and believe that I have an appropriate level of expertise to confirm that it is of an acceptable scientific standard, however I have significant reservations, as outlined above.
